# House mouse subspecies do differ in their social structure

**DOI:** 10.1002/ece3.9683

**Published:** 2022-12-28

**Authors:** Ondřej Mikula, Miloš Macholán, Ľudovít Ďureje, Zuzana Hiadlovská, Kristina Daniszová, Kateřina Janotová, Barbora Vošlajerová Bímová

**Affiliations:** ^1^ Laboratory of Mammalian Evolutionary Genetics, Institute of Animal Physiology and Genetics Czech Academy of Sciences Brno Czech Republic; ^2^ Institute of Vertebrate Biology Czech Academy of Sciences Research Facility Studenec Brno Czech Republic; ^3^ Department of Botany and Zoology, Faculty of Science Masaryk University Brno Czech Republic

**Keywords:** demes, *M. m. domesticus*, modularity, *Mus musculus musculus*, radio‐frequency identification, social networks

## Abstract

It is widely acknowledged that population structure can have a substantial impact on evolutionary trajectories. In social animals, this structure is strongly influenced by relationships among the population members, so studies of differences in social structure between diverging populations or nascent species are of prime interest. Ideal models for such a study are two house mouse subspecies, *Mus musculus musculus* and *M. m. domesticus*, meeting in Europe along a secondary contact zone. Though the latter subspecies has usually been supposed to form tighter and more isolated social units than the former, the evidence is still inconclusive. Here, we carried out a series of radiofrequency identification experiments in semi‐natural enclosures to gather large longitudinal data sets on individual mouse movements. The data were summarized in the form of uni‐ and multi‐layer social networks. Within them, we could delimit and describe the social units (“modules”). While the number of estimated units was similar in both subspecies, *domesticus* revealed a more “modular” structure. This subspecies also showed more intramodular social interactions, higher spatial module separation, higher intramodular persistence of parent–offspring contacts, and lower multiple paternity, suggesting more effective control of dominant males over reproduction. We also demonstrate that long‐lasting modules can be identified with basic reproductive units or demes. We thus provide the first robust evidence that the two subspecies differ in their social structure and dynamics of the structure formation.

## Graphical abstract

In social animals, social structure is strongly influenced by relationships among the population members, so studies of differences in social structure between diverging populations or nascent species are of prime interest. Here, we carried out a series of radio‐frequency identification experiments in semi‐natural enclosures to gather large longitudinal data sets on individual house mouse movements. We provide the first robust evidence that two mouse subspecies differ in their social structure and dynamics of the structure formation.

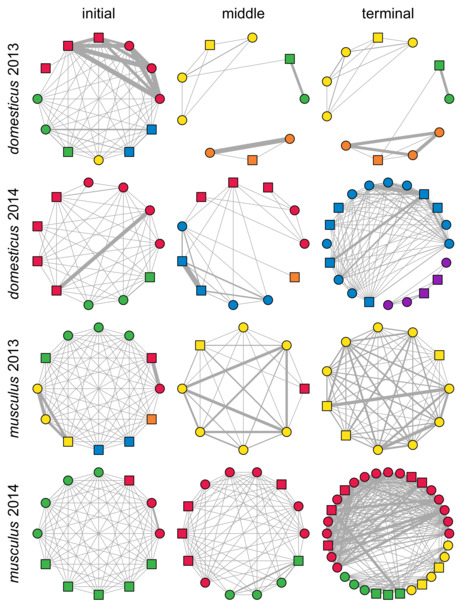

## 1 INTRODUCTION

Knowledge of population structure is crucial for understanding many evolutionary phenomena, including the relative importance of genetic drift and selection (Wright, 1931), adaptation (Kemppainen et al., 2021; Kryvokhyzha et al., 2016; Vahdati & Wagner, 2018), dispersal (Clutton‐Brock & Lukas, 2012; Slatkin, 1987; Stenseth & Lidicker Jr, 1992), the spread of pathogens (Lopes et al., 2016; Sattenspiel, 1987; Thrall & Burdon, 1997), mating patterns (Evans, Lindholm, & König, 2021, 2022; Ferrari et al., 2019; Jarne & Städler, 1995; Odden et al., 2014), or speciation (Coyne & Orr, 2004). In social animals, populations are strongly affected by social interactions and relationships among their members, i.e., their social structure (Kappeler & van Schaik, 2002). In some groups, especially mammals, these interactions are further intensified by physical and nutritional relationships between mothers and their offspring. Trade‐offs between competition and cooperation may interplay in various ways with environmental conditions, resulting in different levels of group cohesion across taxa (Drobniak et al., 2015; Kramer & Meunier, 2019). If parental or communal care increases fitness (König, 1994; Rymer & Pillay, 2018), an evolutionary pathway for more complex sociality may be opened. Moreover, populations can be influenced by a particular mating system. Social and mating systems are thus coupled (Dewsbury, 1990; Kappeler, 2019). For example, small groups usually comprise a single breeding male monopolizing copulations with several adult females. Such units tend to be, to a large extent, isolated from other subpopulations. This isolation may be further strengthened if several group members participate in defense of its territory. By contrast, insufficient control over reproduction by dominant males can result in less close groupings, with extended male–male contests potentially leading to changes in female mating strategies (Clutton‐Brock, 2017).

Differences in social structure can exist even between closely related species (e.g., Stone et al., 2010). However, whether these differences drive the formation of reproductive barriers between nascent species or are a consequence of the isolation is unclear. It is, therefore, necessary to study diverging populations within a single species, just displaying some degree of differentiation in their social structure.

An excellent model for such a study seems to be two house mouse subspecies, *Mus musculus musculus* and *M. m. domesticus*, which diverged ~500,000 years ago (Geraldes et al., 2008; Macholán et al., 2012). These are known to differ in several behavioral traits that can potentially affect population structure, such as higher aggressiveness of *M. m. domesticus* (Ďureje et al., 2011; Piálek et al., 2008; Thuesen, 1977; van Zegeren & van Oortmerssen, 1981) and higher choosiness of *M. m. musculus*, preferring consubspecific mates (Bímová et al., 2005; Smadja et al., 2004; Vošlajerová Bímová et al., 2011). *Mus m. domesticus* males showed longer primary risk assessment but a more active exploration of an unfamiliar space (Hiadlovská et al., 2013; Vošlajerová Bímová et al., 2016) than *musculus* males. By contrast, *musculus* males performed better in challenging situations (Hiadlovská et al., 2014) and were less stressed by handling (Daniszová et al., 2017). More importantly, *domesticus* males appear to establish a social hierarchy faster, which leads to reduced stress, while the social tension tends to persist much longer in *musculus* males (Hiadlovská et al., 2015). Higher social stress can be the reason why subordinate *musculus* males leave their homes more often than dominant males, whereas the opposite holds for *M. m. domesticus* (Hiadlovská et al., 2021).

On the other hand, despite dozens of studies over eight decades of research (see Berry, 1981; Boursot et al., 1993; Krasnov & Khokhlova, 1994; Sage, 1981), our knowledge of ecology and population structure in each mouse subspecies is still equivocal (Ganem, 2012). Mouse populations are generally considered to be subdivided into small, more or less isolated units, usually called demes. Typically, these demes consist of one dominant male monopolising reproduction, several subordinate males, and females with their juvenile offspring. Females can move freely within the deme's territory, whereas subordinate males are usually forced to stay on its periphery. Upon reaching maturity, young males are often coerced into leaving the group, whereas females usually stay and reproduce within the natal deme (see, e.g., Vošlajerová Bímová et al., 2016 and references therein). However, the mouse population structure may vary depending on ecological, climatic, or density situations (Butler, 1980; Noyes et al., 1982; Pocock et al., 2004; Singleton & Krebs, 2007). For example, feral male mice on islands and elsewhere are known to defend individual, exclusive territories that usually overlap with those of several females (Berry, 1970; Krasnov & Khokhlova, 1994; Sage, 1981). Moreover, mice can switch from strong territoriality to a gregarious life during population outbreaks (Singleton & Krebs, 2007). Similar flexibility conditional on or triggered by external conditions has also been described in other species, such as dunnocks (Davies, 1992), African striped mice (Schradin et al., 2012), and primates (Kappeler & van Schaik, 2002). In the house mouse, the ecological and social plasticity is largely associated with the level of its commensal bond with humans. In any case, there is a widespread notion that *M. m. domesticus* is more strictly commensal and hence more “demic” than *M. m. musculus*. However, as Ganem (2012) pointed out, this has not ever been appropriately documented.

This study tested whether the subspecies differ in their social structure using a combination of radio‐frequency identification (RFID) and parentage analysis in a series of longitudinal semi‐natural breeding experiments. To control for the possible influence of different external conditions, we ran the *musculus* and *domesticus* experiments simultaneously, and these parallel runs were repeated for two consecutive years to increase the robustness of the results. The movement data from each experimental run were summarised in the form of social networks.

The structure of time‐extended social networks can be analyzed in various ways (Finn et al., 2019; Holme, 2015). Typically, the whole time of network existence is discretised into periods or time layers and analyzed as a collection of layer‐specific networks. For this purpose, we used a recently published multilayer adaptation of map equation module detection (Aslak et al., 2018), which has several advantages over other approaches since it explicitly estimates the modules as multi‐layer ones. The identity of modules in different time layers need not be assessed post hoc, which is necessary when analyzing such data separately, layer by layer (Evans, Lindholm, & König, 2021; Liechti & Bonhoeffer, 2019). In this way, we could, for the first time, quantitatively demonstrate differences between the subspecies in the social structure of their commensal populations and the dynamics of its formation under semi‐natural conditions.

## 2 MATERIALS AND METHODS

### 2.1 Mice

Founder animals were the first‐generation offspring of reciprocal intrasubspecific crosses between wild mice captured at two *M. m. musculus* localities (Buškovice: 50°13′18″N, 13°22′13″E; Vrbice: 50°8′58″N, 13°13′51″E) and two *M. m. domesticus* localities (Straas: 50°10′54″N, 11°45′57″E; Ottmannsreuth: 49°53′27″N, 11°37′4″E), far enough from the hybrid zone between the subspecies. The wild animals were live trapped in 2012 and 2013, transported to the breeding facility (see Ethics for details), and got rid of ectoparasites and endoparasites. The founder individuals were weaned at 20 days and then kept either separately or with a littermate of the same sex, singly housed later, at 55 days. They were introduced to the arenas as fully grown adults around 90 days of age.

### 2.2 Semi‐natural enclosures

The source data come from semi‐natural enclosures inspired by Perony et al. (2012) and König et al. (2015). Two collateral experiments in separated but neighboring cabins ran for two consecutive years (2013 and 2014), each with *M. m. musculus* and *M. m. domesticus* individuals populating separate enclosures. Each enclosure was seeded with twelve adult founders, six females, and six males. The length of the experiments (in days) was 211 (*domesticus* 2013), 253 (*musculus* 2013), 272 (*domesticus* 2014), and 265 (*musculus* 2014).

Two rectangular enclosures (2 × 4 m each) were partitioned with several polycarbonate panels into six similarly large compartments, each equipped with a nest box and food and water station (Figure S1). In the last phase of the 2014 experiments (195th day since the start), the enclosures were interconnected using two large polycarbonate tubes. The arena floors were covered with ca. 2 cm of wood shavings, and the shredded paper was provided as nesting material. Food and water were available ad libitum during the whole experiments, controlled and refilled every 3 days. Mice were kept under constant conditions (ca. 20°C, 14:10 light–dark cycle). Each enclosure was connected with a single exit box separated by two successive water pools (each 53 × 35 cm with 13 cm deep water) to reduce the social stress of the animals. If an animal remained in the exit box without any detected return to the arena for at least 3 days, it was considered an emigrant and removed from the experiment. The only exception was made when the population in one of the experiments declined drastically, and one pair was allowed to stay in the exit box and reproduce there to serve as a backup in case the main population would get extinct.

Every mouse was injected with a RFID micro‐transponder and marked by toe clipping. Individual movements in and out of the nest boxes were monitored using a pair of transponder readers placed at the entrance to each box. Two readers were also positioned at each side of the tube connecting the two arenas. Moreover, one reader was put on the tube connecting the exit box with the water pool. Data from the readers were continuously collected throughout the whole duration of each experiment. The enclosures were regularly overviewed during minor checks every 3 days and major checks every 30–40 days. In the course of minor controls (taking up to 1 h), we downloaded and back upped the data from data loggers, refilled food and water containers, and checked nest boxes for the presence of litter. All newly born pups were tattooed, sexed, and marked with toe clipping at age ≥7 days. Pups older than 14 days were injected with micro‐transponders. During the major checks that lasted up to 6 h, we also weighed all mice, replaced their transponders if necessary, and collected samples of urine and feces (for additional analyses, not covered by this paper). The enclosures and equipment were cleaned and washed with bleach or 70% EtOH between the experiments.

### 2.3 Statistical processing of data

#### 2.3.1 Preprocessing of the movement data

We defined a visit to a nest box as the time between entering it (recorded as a twofold signal: the first from the outer reader followed by that from the inner reader) and leaving it (recorded in the reverse order, i.e., the inner → outer reader). We cleared the record from equivocal signals, retaining only the credible visits. The record was then divided into discrete time layers. Ideally, these should be of equal length, but enclosure checks made the record semi‐continuous with gaps that were just approximately equally spaced and long. Therefore, we set criteria ensuring the record is not biased by the checks but keeps as much information as possible, and the time layers are long enough to allow a description of the social structure but short enough not to encompass major changes in the structure. First, we ignored the records made during and after enclosure checks (till midnight of the concerned day). Then, we defined the layers as the record fragments no longer than 48 h and involving no gap exceeding 1 h. Finally, we merged the layers shorter than 24 h with their nearest layers. The resulting time layers varied in length (24 to 107, although usually <72 h between the first and the last entry), but each of them could provide a snapshot of social structure. The position of the layers along the time axis corresponds to their mid‐times (in units of days since the start of the experiment). For the assessment of movement between the subspecies/enclosures (2014 runs, see above), we considered as proven all those movements, which were recorded by a reader from one enclosure followed by a record from the other enclosure. Note that a record from a reader does not imply box entrance but still proves presence in the enclosure.

#### 2.3.2 The social network in a single time layer and its structure

We expressed a social contact between two individuals within a single time layer as the total time they spent together in any of the nest boxes, irrespective of other individuals potentially present therein. If $N_{m}$ is the total number of individuals present in the enclosure during day m, the overall daily summary of pairwise social contacts is represented by the $N_{m}\times N_{m}$ matrix with zero diagonal. This can be interpreted as an adjacency matrix (A) of a weighted graph, whose vertices are individuals and undirected edges are interactions between them. The presence of an edge between vertices i and j is indicated in the matrix by a positive value of the element $A_{\mathrm{ij}}$ (or, equivalently, $A_{\mathrm{ji}}$). This value itself represents the weight of the edge, expressing the strength of the interaction. In biological terms, we may interpret this graph as a social network.

As noted above, an observed social network may show some degree of regularity in the arrangement of its edges, which enables describing its structure more concisely. If vertices form clusters whose members are connected more often to each other than to other vertices, we can simplify the network's description by partitioning the vertices into a comprehensive set of nonoverlapping clusters. The map equation introduced by Rosvall and Bergstrom (2008) calculates the description length of a given network. The network structure represents a random variable, which can be thought of as a sequence of vertices visited in a stochastic walk along the edges. Then we can describe the sequence by binary numbers playing the role of codewords denoting vertices visited during the walk. The codewords are chosen in a parsimonious way so that shorter ones are reserved for more frequently visited vertices. The less regular the structure, the more specific and hence longer codewords are required for its description. The average codeword length (L), in the bit units, is thus a suitable measure of the description length. The map equation provides a basis for optimal network partitioning, which is carried out through the minimization of L as described in detail in Rosvall and Bergstrom (2008) and Rosvall et al. (2009).

The clusters of vertices corresponding to the optimal partitioning are called “communities” or “modules” in the social network literature. Modularity M is then a quantity expressing the tightness and exclusiveness of the estimated modules. A new module is created in the map equation framework only if it helps to describe the network structure more concisely. Hence, modularity can be quantified using the average codeword lengthL, but this value is dependent on the network size. Therefore, we define M as a compression rate of L, i.e., the ratio of its value before and after partitioning into the modules:

$$M=\frac{L_{\text{before}}}{L_{\text{after}}}.$$

In practice, optimal partitioning is found using the Louvain algorithm (Blondel et al., 2008) with L as the objective function. The search is not constrained in any specific way, and the modules can thus be of any size and composition; it only matters if their delimitation reduces L. Note that our definition of modularity is different from the most common use of the term (Newman & Girvan, 2004). We use the word as a general term expressing the degree of partitioning into modules.

#### 2.3.3 Time dimension

Social relations may change over time, for example, due to birth, death, and migration changing population size and composition. We created a time‐ordered collection of layer‐specific social networks for each of the four experimental runs. Then we estimated time‐extended modules under the extension of map equation formalism described by Aslak et al. (2018) and implemented in the program Infomap (Rosvall & Bergstrom, 2008). In this algorithm, vertices are linked to their neighbors within the same layer as well as with the same set of neighbors in all other layers. The weights of these cross‐layer links depend on the similarity of neighborhood patterns in two particular layers. The cross‐layer links are strong if a vertex has the same neighbors and similar edge weights in both layers. The algorithm then clusters vertices across the layers with the probability dependent on the weight of cross‐layer links and a tuneable relaxing rate parameter $r\in \left\langle 0,1\right\rangle$ which balances the importance of within‐layer and cross‐layer links. The clustering criterion is still the same, L, and so is the definition of modularity M. Every individual is assigned to a single module in any particular time layer, but the assignment may change between time layers. Choosing the relaxing rate and the randomization procedure used for assessing the robustness of the differences in modularity are described in more detail in Supplementary Material. Finally, we examined the sex‐specific roles in the social structure dynamics. We re‐calculated *M* in each experiment from only female–female, male–male, or female–male interactions while keeping the clustering of individuals fixed to the estimated solution. In these calculations, we included only adults (≥50 days old individuals).

For illustrative purposes, we depicted three selected social networks from each experiment. The selection was motivated by the observed results (see below). Specifically, we used the networks from (i) the initial period when most of the founders were still present (the first 10 time layers); (ii) 10 time layers covering the period after the establishment of new major modules (starting 30 days after their appearance); and (iii) the last 10 time layers of the experiment when the population structure can be considered “mature”. For this display, we calculated the mean interaction strength in the time layers involved. Only the founder and/or already reproducing individuals were included in the calculation (reproduction being proven by parentage analysis, see below).

The estimated value of modularity depends on the relaxing rate (*r*), which has to be chosen in some principled (nonarbitrary) way. Our focus was on the most robust features of social structure; hence, we chose the value whose solutions were most similar to solutions produced under all other relaxing rate values. We tried 21 *r* values (equally spaced between 0 and 1). In each experiment, we used normalized mutual information (NMI; Strehl & Ghosh, 2002) to quantify pairwise similarities of solutions corresponding to particular relaxing rates and evaluated each *r* by its mean NMI. The consensual *r* was chosen to maximize the median of experiment‐specific mean NMIs.

We also employed a bootstrap procedure to assess whether the differences in modularity are robust to the RFID record's idiosyncrasies. Bootstrap replicates were created by random reordering of nest visits in the movement records of every individual. Therefore, even in bootstrapped data, every individual spends the same amount of time in the same nest boxes as in the observed data, but its pairwise associations are disturbed. More affected should be those established by just a few moderately long encounters, while associations based on frequent and repeated encounters are likely to be recovered even after randomization. Modularity was calculated under all 21 relaxing rates for the observed and hundred bootstrapped data sets.

#### 2.3.4 Spatial dimension

In time layer summaries and all other analyses described above, the location of encounters was ignored. Nevertheless, once the network is partitioned, we can work backward and detect where the identified modules were settled. Separately for each layer, we calculated the total amounts of time spent by members of particular modules in each of the six boxes. These times were divided by the total usage of the box, which is the sum of times spent by all individuals in the box. The resulting proportions can be understood as box possession values, and their maximum was taken as a measure of the box usage exclusivity. Then, for a given time layer, we calculated an index of spatial separation as a weighted mean of box usage exclusivities, weights being proportional to the total usage of the boxes.

The connection of enclosures brought an extra spatial dimension to the two 2014 parallel runs. Therefore, we examined how often mice moved between the enclosures (evidenced by any signal from the opposite side) and whether they participated in the other subspecies' social network (evidenced by box entrances).

#### 2.3.5 Software

Apart from Infomap (Rosvall & Bergstrom, 2008), all the statistical analyses described above were implemented in the computing language and environment R (RCore Team, 2019). A collection of functions performing the core of our analyses and drawing the main figures is available at the publicly available repository (https://github.com/onmikula/movement_networks). Included is also a worked‐out example based on *domesticus* 2013 experiment. The scripts relied on the functionality of the packages igraph (Csardi & Nepusz, 2006), Matrix (Bates & Maechler, 2019), abind (Plate & Heiberger, 2016), and stringr (Wickham, 2019). For plotting, we used packages vioplot (Adler & Kelly, 2018) and TeachingDemos (Snow, 2020). The color palette used for the module display was designed by A. Trubetskoy (https://sashamaps.net/docs/resources/20‐colours).

### 2.4 Parentage assignment and inference of demes

We collected tissue samples from founder mice and all their descendants for subsequent parentage analysis. DNA was then extracted from these ethanol‐preserved tissues using DNeasy 96 Blood & Tissue Kit (Qiagen) following the manufacturer's instructions. Panels of 25 and 26 microsatellites were used for parentage assignment (see Table S1). PCR conditions consisted of an initial denaturation at 95°C for 15 min, followed by 30 cycles of 94°C for 30, 90 s at a specific annealing temperature (see Table S1), and 72°C for 60 s. A final extension step at 60°C lasted 30 min. PCR products were then analyzed on an ABI3130XL Sequencer and visualized using GeneMarker v. 1.95 (http://www.softgenetics.com).

Parentage analysis was performed using CERVUS v. 3.0.3 (Kalinowski et al., 2007) at a 95% confidence level. Individuals meeting defined requirements (e.g., reaching sexual maturity at the estimated time of conception) were included in the analysis as candidate parents. Based on these data, we assessed the reproductive success of all males and females during the whole experiment.

We quantified multiple paternity for each experimental run as the mean probability that two offsprings from the same litter have different fathers. We also examined the association between modular structure and reproductive behavior. Specifically, we asked to what degree reproduction took place within modules. For this purpose, mating was assumed to occur 20 days before delivery, and we checked if the parents were members of the same module at that time. Then we assessed whether the offspring remain in the same module as their parents or whether they emigrate. It was accomplished by constructing parentage networks with arrows leading from parents to their offspring. We calculated the persistence of family bonds as the proportion of the parentage arrows found within modules for each time layer and each experimental run. These statistics were also evaluated specifically for mother–daughter and father–son interactions.

Parentage data also allow us to address one of the principal issues of this study, i.e., whether and how the inferred social network modules can be related to basic reproductive units or demes. Our approach to the identification of putative demes is based on the premise that a social network module corresponds to a deme if at least one parent pair produced its offspring when it was strongly associated with this module. Any parent pair can support the demic nature of the module to which the mother belonged at the estimated time of conception. This support is expressed as the proportion of time layers in which both parents were associated preferentially with the module where the offspring were conceived. The following example can illustrate its calculation.

Consider a single pair, female Nora and male Miro, who delivered a progeny at time layer no. 15. The estimated time of conception falls into time layer no. 6 when Nora was associated with the “red” module. Hence, Nora and Miro can thus provide support for the “red” module being a deme. In this case, the period from conception to delivery is covered by 10 time layers. If both parents were associated preferentially with the “red” module in all ten layers, the support (strength of the evidence) would be 1.0. On the other hand, if the association were limited to only two time layers, the support would be 0.2. These values were calculated for every litter of every parent pair and then summed up. We can thus regard the final sum as the effective number of parental pairs whose reproductive behavior is socially associated with a given module. This means that if one pair twice conceives a progeny and spends the whole time of gravidity in one module, the final effective number of pairs is two. Conversely, two pairs with a half association with the module are counted as one. Zero strength implies social bonds within the module cannot be unequivocally identified with reproductive relationships, and hence we cannot consider the module to be a deme.

## 3 RESULTS

### 3.1 Populations

**FIGURE 1:**
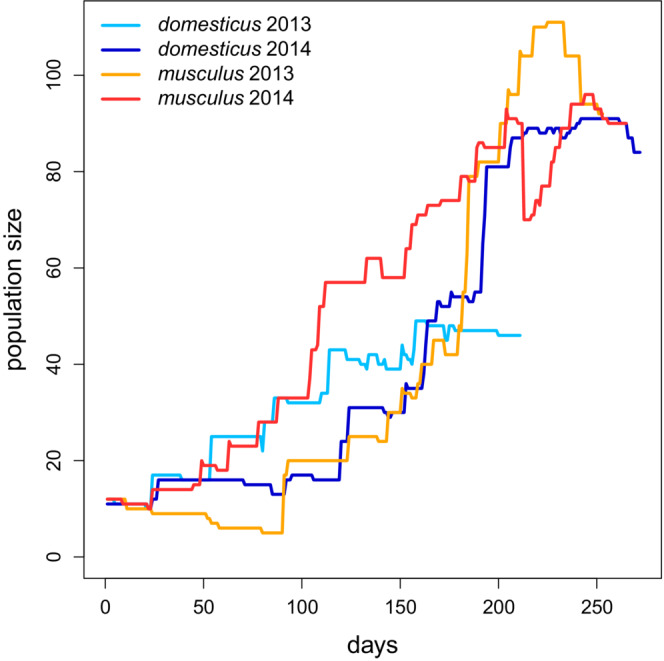
Population changes during four experiments. On the abscissa are days since the beginning of each run. On the ordinate are the total numbers of individuals present in the enclosure.

All but one of the experimental runs revealed a similar trend in population growth. The only exception was the “*musculus* 2013” population in which a severe decline preceded the growth period (Figure 1; see also Figure S2). Maximum population sizes were 49 in *domesticus* 2013, 91 in *domesticus* 2014, 111 in *musculus* 2013, and 96 in *musculus* 2014. There were usually only 1–2 (occasionally up to five) pregnant or nursing females at the same time until the 150th day. After that, there were large fluctuations in all runs (Figure S2c,d). Table S2 shows details on the total number of individuals, the numbers of those that reached adulthood and sexual maturity, as well as those participating in reproduction. Reported is also the number of delivered offspring and the number of litters. (Note that while the numbers shown in the figures refer to individuals involved in RFID‐tracked interactions, those in the tables refer to all registered individuals.)

### 3.2 Modules

The analysis integrating multiple time layers revealed 4–11 modules in each experiment. The modules varied greatly in their size and lifespan, some comprising a few isolated individuals and lasting no longer than one or a couple of days. The number of long‐lasting modules (>50 days) was just 3–5 (see Table S3 for an overview). The number of modules was not dependent on the population size in any obvious way, but new modules sometimes arose with the introduction of new offspring, i.e., with the appearance of new vertices. This is because the algorithm does not explicitly consider the appearance of new vertices or the disappearance of old ones, and a sudden significant change in population composition thus results in the introduction of new modules to minimize the description length.

**FIGURE 2:**
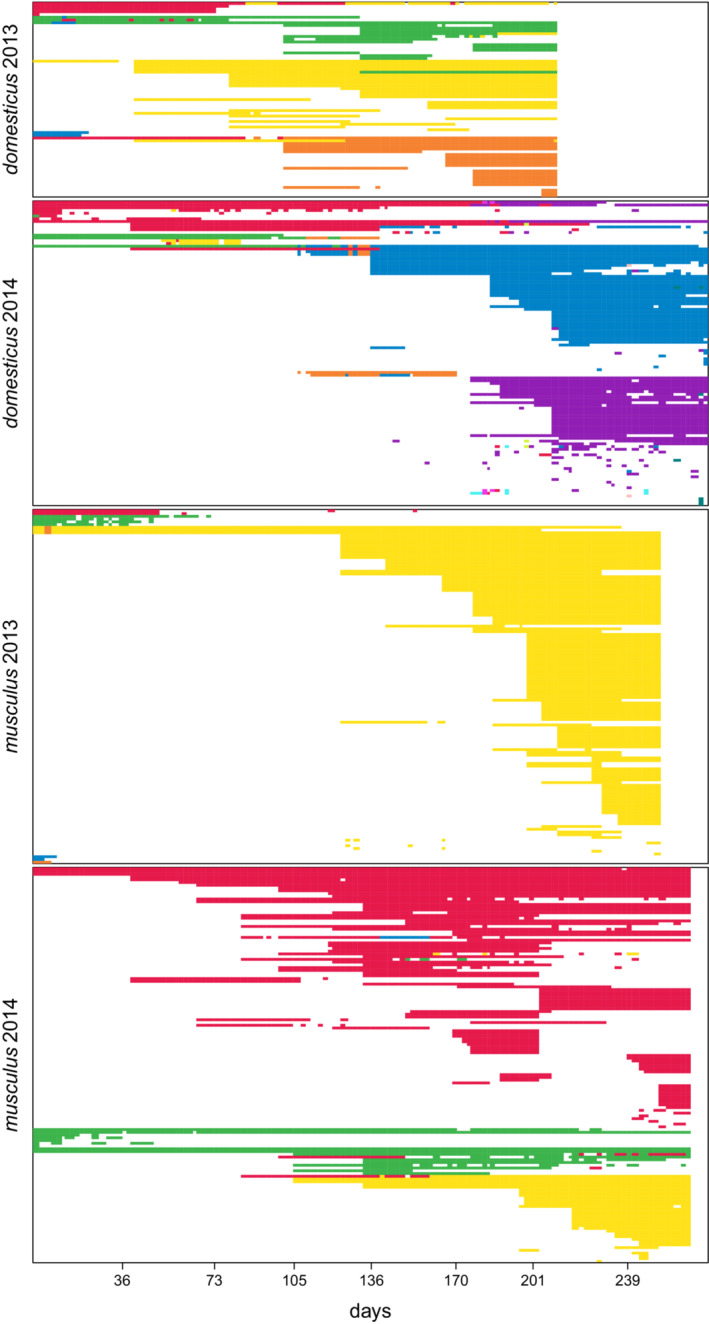
The modular structure of the experimental populations. Rows of the bar plots correspond to individuals and columns to time layers. On the abscissa is time in days. Colors indicate module membership, while blank spaces indicate time layers in which the individuals did not enter any box. Note that in the “musculus 2013” experiment, the red module persists considerably long via the episodic occurrence of a single “red” individual. This is the trace of a subpopulation allowed to survive in the exit box (see Supplementary Material).

The multilayer modular structure is graphically depicted in Figure 2. Each row corresponds to a single individual in this figure, while each column represents one time layer; assignments of individuals to modules are shown in different colors. Immediately after launching the *domesticus* 2013 experiment, four modules were formed, but one of them (“blue”) survived only until the 21st day, and another (“red”) dissolved into “yellow” and “orange” modules, respectively. The latter was estimated to form at about the 104th day when the offspring of the “red” module's founding female were weaned, received transmitters, and thus entered the record. In the *domesticus* 2014 run, two modules that arose initially (“red” and “green”) were gradually transformed into two modules dominating the social structure at the end of the experiment (“blue” and “purple”). The transformation reflected a gradual extinction of founding individuals and their replacement by newly born offspring. In addition, there were seven modules, often representing a temporary association of siblings with some of their parents or an episodic interaction of immigrants with local inhabitants. The *musculus* 2014 run started with two modules (“red” and “green,” both persisting until the end), from the 106th day on, accompanied by the third module (“yellow”). In both persisting initial modules, three of their founders also survived until the end, whereas the later‐appearing one was established by the first‐generation offspring born in the enclosure. The fourth module, which also appeared in this experiment, was marginal. Finally, as noted above, the *musculus* 2013 run was very different from the remaining experiments. While five modules occurred initially, two of them were substantially short‐lived, and only one module (“yellow”) survived, dominating the rest of the experiment.

### 3.3 Modularity

**FIGURE 3:**
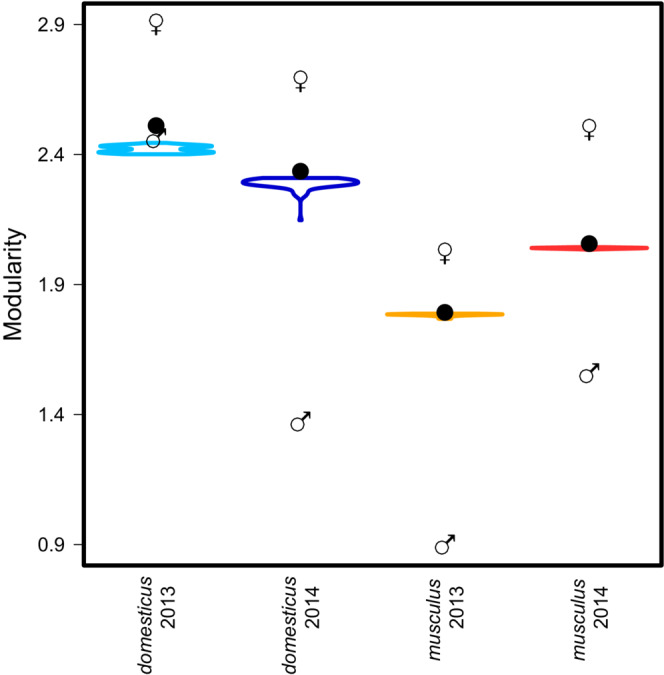
Modularity quantified for each experimental run as the compression rate of the description length (see Section 2 for details) compared between original data (black dots) and randomized replicates (violin plots). Venus and Mars's symbols show values based on female–female and male–male interactions, respectively.

To quantify the degree of tightness and exclusiveness of the estimated modules, we introduced a new index of modularity, *M* (see Section 2 for details). The most fundamental result of our study is the difference in this index between the subspecies. While in *M. m. musculus*, *M* was 1.78 (2013) and 2.05 (2014), respectively, in *M. m. domesticus*, it was as high as 2.51 (2013) and 2.33 (2014), respectively (Table 1). Figure 3 shows these estimates together with the randomization support (represented by violin plots). The difference between subspecies is apparent even in the ordination of randomized distributions. The modularity of *M. m. domesticus* populations seems to be slightly more dependent on individual movement details, but it still holds that their minimum randomized *M* was higher than that observed in *M. m. musculus* populations (Table S2). In all cases, *M* values calculated from female–female interactions were considerably higher than those based solely on male–male interactions within individual runs (see gender symbols in Figure 3). By contrast, *M*s calculated from male–female interactions (Table S2) were all close to the overall values. The presented solution was obtained with the relaxing rate parameter *r* = 0.60. However, the full sequence of *M* values obtained under different relaxing rates also supports the conclusion that *M. m. domesticus* is more “demic” than *M. m. musculus* (Figure S3) and confirms, via randomization, that differences in *M* are reasonably robust to accidental details of movement records.

**TABLE 1:** Modularity in the four experimental populations, calculated with *r* = 0.60

	Full data	Female–female	Male–male	Male–female	Minimum randomized value	Maximum randomized value
*domesticus* 2013	2.51	2.91	2.46	2.44	2.40	2.44
*domesticus* 2014	2.33	2.68	1.36	2.38	2.14	2.31
*musculus* 2013	1.78	2.01	0.88	1.88	1.76	1.78
*musculus* 2014	2.05	2.50	1.54	2.13	2.03	2.04

**FIGURE 4:**
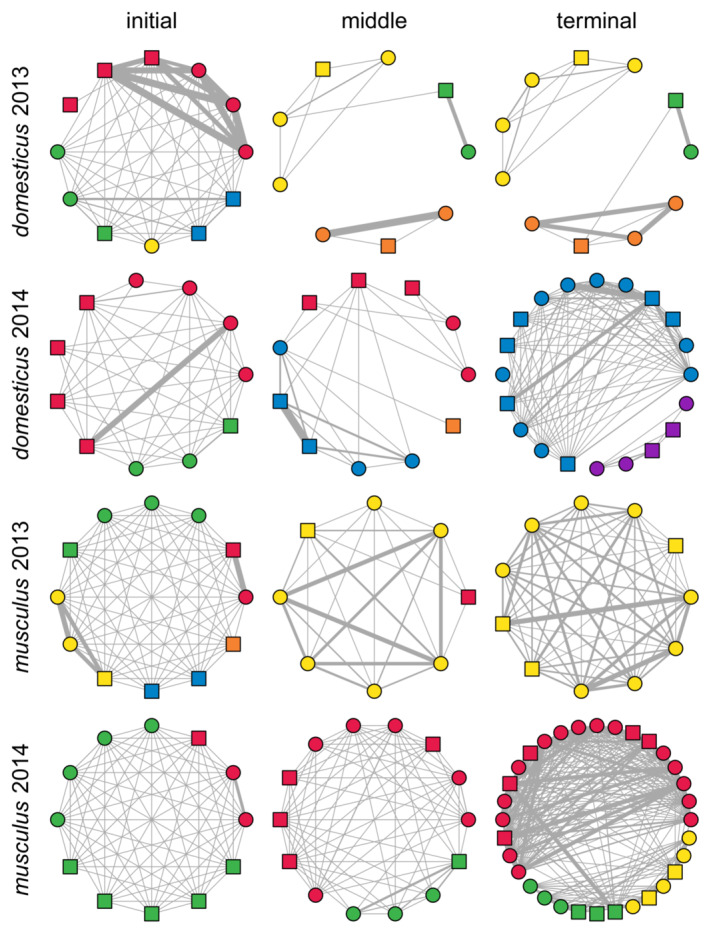
Summary of social networks calculated for three periods, each consisting of ten time layers. Line widths reflect the strength of social interaction; vertex colors indicate prevailing module membership, and their shapes correspond to sex (squares are for males, circles are for females). The networks cover the following time spans (in days since the start of the experiment): 1–25, 136–155, 195–211 (*domesticus* 2013); 1–21, 134–156, 250–272 (*domesticus* 2014); 1–25, 136–155, 233–253 (*musculus* 2013); and 1–21, 134–156, 245–265 (*musculus* 2014). The middle period covered the 61st to 70th time layer in all experimental runs. The displayed networks contain only individuals involved in reproduction.

Summary networks are depicted for the three selected periods: the first ten layers of each experiment (initial), ten layers covering an advanced phase of the demic dynamics (middle), and the last ten layers (terminal). These graphs illustrate the social structure difference between the two subspecies: while *M. m. musculus* displays multiple and recurrent intermodular interactions, these vanish with time in *M. m. domesticus* (Figure 4).

**FIGURE 5:**
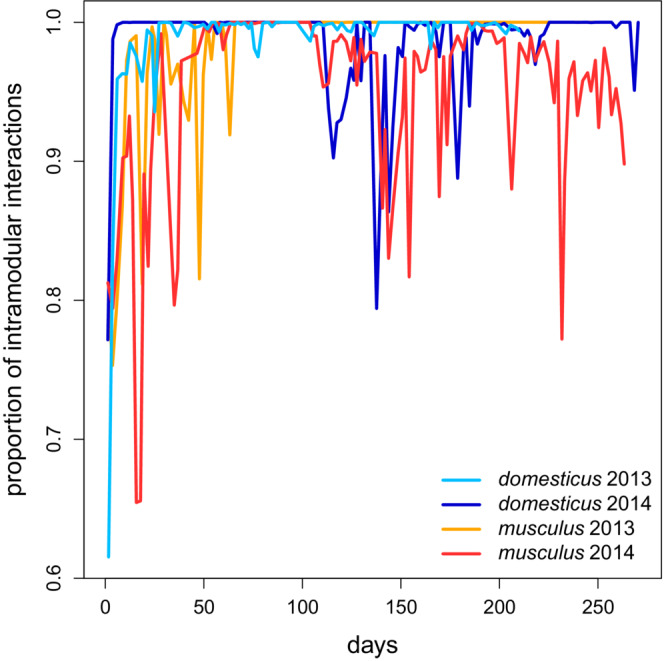
Module distinctiveness quantified as a mean proportion of time spent by individuals in interactions with their module members

The between‐subspecies difference in the level of modularity is corroborated by module distinctiveness expressed as the proportion of intramodular interactions (Figure 5). The maximum value of 1.00 means all individuals met in the boxes only with members of their modules). In both *M. m. domesticus* populations, this proportion approached 1.00 in 5–30 days after launching the experiments and tended to remain close to the maximum value until the end. Even in *domesticus* 2014, occasional deep drops were followed by fast recovery of high distinctiveness. A more detailed examination revealed these drops could be explained by the temporary relaxation of father–son bonds (Figure S6). In *musculus* 2014, module distinctiveness reached its maximum later (after ~50 days), and it remained high only until the rise of the third module (“yellow” in Figure 2) on the 106th day. Since then, it fluctuated between 0.77 and 1.00. Again, loose father–son bonds can be largely responsible for the lower distinctiveness of the modules (Figure S6). From around the 50th day onward, only a single module existed in the *musculus* 2013 population. The mice could only interact within their group, and distinctiveness was 1.00, thus resembling *M. m. domesticus*.

**FIGURE 6:**
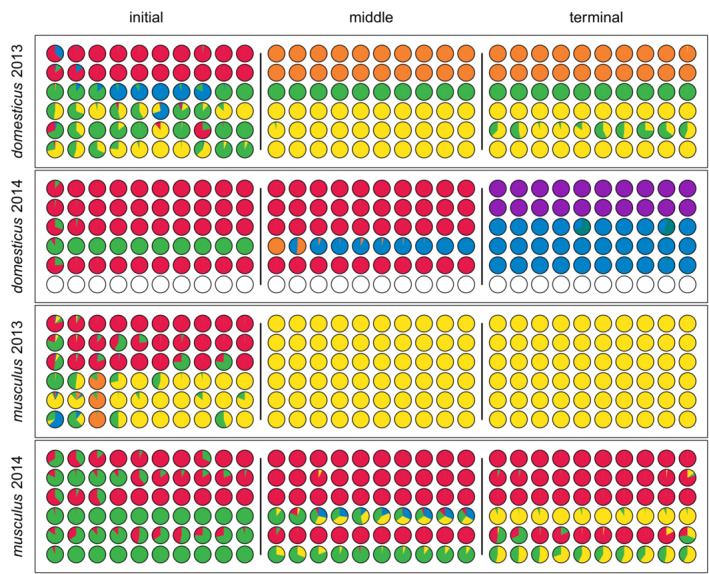
The spatial separation of modules in three selected periods of the four experimental runs. Rows correspond to nest boxes, columns to time layers, and pies show the proportions of box occupancy by members of different modules (with color code as in Figure 2; empty circles depict unoccupied boxes). Every period consists of 10 time layers, the same as in summary networks

The spatial separation of modules (Figure S4) closely parallels the picture revealed by module distinctiveness. These differences are further corroborated if we look at each of the six nest boxes' occupancies by members of individual modules (Figure 6) in the same periods as covered by the summary networks shown in Figure 4. In both subspecies, a substantial spatial admixture can be seen in the initial period. Later on, a visible difference arises. In *M. m. domesticus* runs, the admixture is very limited, confined to a single nest box, a few time layers, and in *domesticus* 2014, also to marginally significant modules. On the contrary, in *M. m. musculus*, the admixture persisted in four nest boxes and was quite extensive, especially between the green module and the rest (in the *musculus* 2014 run, which was the only informative run in this respect, containing more than one module).

### 3.4 Connecting enclosures

Connecting the arenas during the last part of the 2014 experiment resulted in the migration of some individuals to the opposite enclosure. We recorded 266 such events, but 154 of them (58%) were due to just five males: four *domesticus* and one *musculus* male. The remaining 112 migrations were due to 49 individuals (nine females/19 males in *domesticus* and 11 females/10 males in *musculus*), distributed in time as shown in Figure S5. The migration rate was highest shortly after interconnecting the enclosures in both subspecies, yet this remained high much longer in *M. m. domesticus*, with another peak ~50 days after the interconnection, short before the end of the experiment. These remaining migrations were distributed between the subspecies and sexes as follows: 20 in *domesticus* females, 54 in *domesticus* males, 14 in *musculus* females, and 24 in *musculus* males. These numbers are negligible compared to thousands of movements recorded within every time layer in both enclosures, but on the other hand, they indeed underestimate real figures. Due to design limitations (just one reader on each side of the connecting tube), it was hard to distinguish real migration from the background of unrealised migration attempts. In total, we detected as many as 3813 attempts, but we do not know for sure how many of them were successful. Interactions of the migrants with residents were scarce. First, we did not find their co‐occurrence within a single nest box. And second, although five *musculus* individuals were occasionally involved in *domesticus* networks and one *domesticus* individual entered *musculus* boxes, all these cases were detected within just a single time layer shortly after interconnecting the arenas. In no case, the intruders stayed and reproduced in the opposite enclosure.

### 3.5 Parentage and demic structure

**FIGURE 7:**
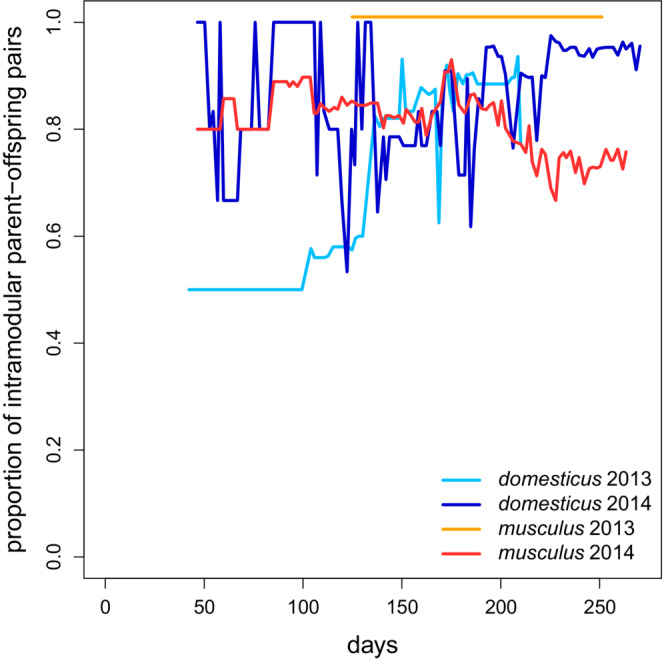
The persistence of family bonds within modules shown as changes in the proportion of parent–offspring pairs found within modules through time

In *M. m. domesticus*, the offspring appears more likely to remain with their parents in the same module (Figure 7). Sex‐focused analyses reveal that this is mainly due to mother–daughter interactions (Figure S6). Multiple paternity (i.e., probability that two siblings have different fathers) was equal to 0.04 in *domesticus* 2013, 0.15 in *domesticus* 2014, 0.11 in *musculus* 2013, and 0.42 in *musculus* 2014. Note that the value in *musculus* 2013, where a single module was present for most of the trial duration, is almost as high as in *domesticus* 2014. It is also striking because the time window when this multiple fathering came about was relatively narrow—reproduction started with just one male surviving in the enclosure, and the first offspring sired by someone else appeared as late as 94 days after the first offspring male was born. These results suggest looser intramodular bonds and/or lower control of a dominant male over reproduction in *M. m. musculus* relative to *M. m. domesticus*.

Knowledge of associations between parents, their offspring, and the modules they are mostly associated with allows us to relate individual modules to reproductive units (demes), i.e., inferring the number of presumed demes in each experiment. Overall, inferred demes perfectly matched the long‐lasting modules persisting for more than 50 days. Support for individual demes ranged from 0.92 (“red” module in “*domesticus* 2013”) to 33.04 (“red” module in “*musculus* 2014”; cf. Figure 2 and Table S3). All other modules could not be considered true demes.

## 4 DISCUSSION

In all experimental runs, *M. m. domesticus* displayed higher modularity than *M. m. musculus* (with the difference ranging from 0.28 between the “*domesticus* 2014” and “*musculus* 2014” populations to 0.73 between the “*domesticus* 2013” and “*musculus* 2013” population). It means that the former subspecies has a closer population structure, which can involve either subdivision into modules or a higher degree of regularity in intramodular interactions (recall that module tightness is defined in terms of compressibility in the map equation framework, which is unrelated to internal link density).

Since we could run only two replicates of RFID experiments per subspecies, we could not test whether the contrast in modularity between them was significant. However, there are reasons to believe the difference is real. First, there is a clear difference in the distinctiveness of the estimated modules (Figure 4). In *M. m. domesticus*, their boundaries were very sharp, with little or no contact between members of different modules. In contrast, contacts between different modules' members were commonplace in *M. m. musculus* (cf. Figures 4 and 5). Second, within *M. m. domesticus* modules, nest boxes were shared much less often than in *M. m. musculus*. Although sharing a nest box does not always mean a direct encounter, higher versus lower spatial separation indicates a real behavioral/ecological difference (Evans, Liechti, et al., 2021). Third, more intramodular parent–offspring pairs in *M. m. domesticus* than in *M. m. musculus* (Figures 7 and S6) are consistent with the higher modularity of the former subspecies. Finally, multiple paternity was considerably lower in *M. m. domesticus*, suggesting higher dominance and control over reproduction in this subspecies. This contrasts with previous studies reporting comparable or only slightly lower multiple paternities in *M. m. domesticus* (Dean et al., 2006; Firman & Simmons, 2008; Thornhauser et al., 2014; our unpublished data).

Although this study focuses on contrasting *musculus* and *domesticus* in the first place, we should also note higher modularity in females than males within each subspecies (Figure 3). This is consistent with the higher persistence of mothers–daughters bonds than fathers–sons interactions (Figure S6), possibly suggesting higher philopatry of females. Our study thus corroborates the results of Evans, Lindholm, and König (2022), who found that females of *M. m. domesticus* preferentially breed within their maternal community.

Since the 1950s, lots of ecological and genetic studies have suggested that *M. m. domesticus* populations are structured into small and relatively rigid and closed units (Anderson, 1964; Crowcroft, 1955; Crowcroft & Rowe, 1963; Lewontin & Dunn, 1960; Lidicker Jr., 1976; Reimer & Petras, 1967; Selander, 1970) and that these units or demes do not survive longer than a few months (Evans, Lindholm, & König, 2021; König et al., 2015; Pocock et al., 2005; Singleton, 1983). However, considerable evidence has also been gathered that many *domesticus* populations are not so tightly organized (Berry, 1981; Sage, 1981). Nevertheless, this subspecies has generally been considered more “demic” than *M. m. musculus*. Such opinion is based on two tenets: first, the demic structure is characteristic of commensal populations, and second, *domesticus* is often believed to be more commensal than *musculus*. However, as Ganem (2012) pointed out, the latter assumption has never been reliably documented.

Moreover, it should be mentioned that *M. m. musculus* is also ecologically highly flexible, as shown by many studies (Krasnov, 1988; Krasnov & Khokhlova, 1994; Pelikán, 1981; Petrusewicz & Andrzejewski, 1962; Walkowa, 1981). On the other hand, permanent non‐commensal *M. m. domesticus* populations are relatively common (e.g., Cassaing & Croset, 1985; Hardouin et al., 2010; Navarro et al., 1989; Sage, 1981; Triggs, 1991; Webb et al., 1997). In this context, it is important that we seeded all the experimental populations with individuals collected from the same central European area (two within the *M. m. domesticus* distribution area and the other two within the *M. m. musculus* range). The sampling sites are located at similar latitudes and altitudes and represent the same indoor, commensal habitat. This way, we avoided potential confounding effects of different environmental conditions known to affect house mouse ecological strategies (Butler, 1980; Noyes et al., 1982; Pocock et al., 2004; Singleton & Krebs, 2007) and hence demonstrated a significant distinction in the social structure between the two subspecies without any reference to (either real or suspected) differences in the level of commensalism. Likewise, owing to identical conditions in the enclosures, we assume the differences revealed in this study are not simply a manifestation of the social flexibility reported in several bird and mammal species (Davies, 1992; Kappeler & van Schaik, 2002; Schradin et al., 2012).

Given our method of detecting social network modules, one key question arises: How can the identified modules be related to basic reproductive units or demes? We believe this issue can be addressed by combining paternity data with information on associations with individual modules across time layers. Our results show a close relation between demes and stable modules persisting for more than 7 weeks (Table S3).

The higher modularity of *M. m. domesticus* appears consistent with about twice as high global effective population size (*N*_e_) as that of *M. m. musculus* (Geraldes et al., 2008; Phifer‐Rixey et al., 2012; Salcedo et al., 2007). (Interestingly, the Asian subspecies *M. m. castaneus*, considered the most strictly commensal of the three main house mouse subspecies (Sage, 1981), also has the highest *N*_e_ (Geraldes et al., 2008; Phifer‐Rixey et al., 2012). Therefore, it would be useful to extend the experiment reported here also to *castaneus*.) In contrast, socially mediated reduction of gene flow, in combination with polygynous mating, should decrease *N*_e_ of *local* demes more in *M. m. domesticus* than in *M. m. musculus*. Joint effects of genetic drift and inbreeding in small, socially structured subpopulations can facilitate the fixation of underdominant chromosomal rearrangements such as Robertsonian fusions (Britton‐Davidian et al., 2007; Dallas et al., 1998; Nachman & Searle, 1995). According to Bush (1975) and Wilson et al. (1975), this process can result in establishing postzygotic reproductive isolation promoting stasipatric speciation (Sites & Moritz, 1987; White, 1978). However, given the great ecological plasticity of house mice, it is unclear how the differences in social structure between the two taxa evidenced in this study relate to the dynamics of secondary contact between them.

By connecting the enclosures around the last quarter of the 2014 experiments, we wanted to simulate an initial contact between the subspecies and appraise potential differences in their dispersal and exploration strategies. Surprisingly, migrations between the arenas were infrequent, and their frequency was even decreasing with time (Figure S5). This finding may correspond with the poor ability of mice to re‐invade sub‐Antarctic Kerguelen islands already populated by residents (Hardouin et al., 2010). We further corroborated the higher dispersion rate of males in both taxa as well as a more active exploration of an unfamiliar space by *M. m. domesticus* of both sexes (Hiadlovská et al., 2013; Vošlajerová Bímová et al., 2016). However, all the migration events were ephemeral, reflecting the inherent neophilia of house mice (Barnett, 1988; Chitty, 1954), although this may apply more to lab mice than wild mice, as shown by Kronenberger and Medioni (1985) rather than actual dispersal.

To conclude, we showed that describing network structures through binary codeword lengths estimated by the map equation (Rosvall & Bergstrom, 2008) and the vertex‐level coupling method (Aslak et al., 2018) is very useful for treating large RFID‐based longitudinal data sets. This method not only avoids the need for analyzing successive time layers one by one but also separates two conceptually different issues: the quantification of cross‐layer coupling and its weighting relative to intralayer connectivity. While the cross‐layer coupling reflects the similarity of intralayer connectivity patterns, the relative weights are given by choice of some relaxing rate. In contrast, the multilayer generalization of Newman and Girvan's modularity (Mucha et al., 2010) conflates these issues by relying on tunable weights of links connecting identical vertices in different layers. As a result, the persistence of modules through multiple layers is less likely due to the particular choice of the tuning parameter. It is also worth stressing that although modularity defined by the compression rate of the average code length is unbounded at its upper end, it is independent of network size, a favorable property it shares with Newman and Girvan's modularity. Finally, it is expected to suffer less from the resolution limit problem (Kawamoto & Rosvall, 2015), i.e., the impossibility of detecting modules that are too small relative to the overall size of the network (Fortunato & Barthelemy, 2007). In this way, we could render, for the first time, robust quantitative evidence that commensal populations of the two European house mouse subspecies subjected to identical environmental conditions develop diverse social networks and hence differ in their social structure.

## AUTHOR CONTRIBUTIONS

**Ondřej Mikula:** Formal analysis (lead); software (lead); writing – original draft (equal); writing – review and editing (equal). **Miloš Macholán:** Conceptualization (equal); funding acquisition (lead); project administration (lead); supervision (equal); writing – original draft (equal); writing – review and editing (equal). **Ľudovít Ďureje:** Data curation (lead). **Zuzana Hiadlovská:** Data curation (equal); writing – review and editing (equal). **Kristina Daniszová:** Data curation (equal); investigation (equal); writing – review and editing (equal). **Kateřina Janotová:** Data curation (equal). **Barbora Vošlajerová Bímová:** Conceptualization (equal); supervision (lead); writing – review and editing (equal).

## FUNDING INFORMATION

The study was funded with the Czech Science Foundation grants nos. P506‐11‐1792, 19‐19056 S (to MM), and 17‐25320 S (to BVB). Computational resources were supplied by the project “e‐Infrastruktura CZ” (e‐INFRA LM2018140) provided within the program Projects of Large Research, Development and Innovations Infrastructures.

## CONFLICT OF INTEREST

The authors declare no competing interests.

## Supporting information


Appendix S1

## Data Availability

We have put a cleared and polished set of functions performing the core of our analyses and drawing the figures to the publicly available repository (https://github.com/onmikula/movement_networks).
