# Supplementary material for: House mouse subspecies do differ in their social structure
Source: Ecol Evol. 2022 Dec 28;12(12):e9683. doi: 10.1002/ece3.9683 (PMC9797468; doi:10.1002/ece3.9683)
Supplement: Supplementary file 1 — Appendix S1 [file ECE3-12-e9683-s001.docx]

### Supplementary Material

### Methods

### Semi-natural enclosures





**Figure S1.** Schematic depiction of one experimental enclosure.

### Parentage analysis

**Table S1**. The panel of microsatellites used for parentage analysis.

| Multiplex #  (annealing temperature) | Marker^*^ | Dye | notes |
| --- | --- | --- | --- |
| 1  (60°C) | D10Mit274 | VIC |  |
|  | D12Mit4 | NED |  |
|  | D3Mit203 | 6-FAM |  |
|  | D5Mit25 | PET |  |
|  | D6Mit37 | VIC |  |
| 2  (60°C) | D10Mit49 | PET |  |
|  | D15Mit79 | 6-FAM |  |
|  | D17Mit139 | VIC |  |
|  | D1Mit231 | 6-FAM |  |
|  | D1Mit506 | PET |  |
|  | D7Mit318 | NED |  |
| 3  (63°C) | D15Mit239 | NED |  |
|  | D1Mit46 | VIC |  |
|  | D2Mit1 | PET |  |
|  | D2Mit200 | 6-FAM |  |
|  | D5Mit144 | VIC |  |
|  | D9Mit321 | PET |  |
|  | D9Mit96 | 6-FAM |  |
| 4  (58°C) | D13Mit77 | 6-FAM | *Mmd* |
|  | D16Mit55 | NED |  |
|  | D1Mit236 | 6-FAM | *Mmd* |
|  | D2Mit92F | VIC |  |
|  | D4Mit149 | VIC |  |
|  | D4Mit171 | VIC |  |
|  | D4Mit187 | 6-FAM |  |
|  | D7Mit66 | PET | *Mmm* |
|  | M334^†^ | PET |  |

^*^Names of markers and primer pairs are according to the MIT Genome Center (MGI version 6.16; <http://www.informatics.jax.org>); ^†^M334 is presented in Vyskočilová et al. [1]. Dye refers to the fluorescent label for each forward primer. *Mmd* = marker used only for *M. m. domesticus* samples; *Mmm* = marker used only for *M. m. musculus* samples.

## ****Results****

**In all the experimental runs, the animals were breeding successfully. In three out of four runs, after the initial elimination of some founders, first litters were delivered around the 23^rd^ day, and then the population size gradually, although not monotonically, increased with a rapid growth period between the 100^th^ and 150^th^ day of the experiment. The only exception was the** **‘*musculus* 2013’ run, where the first litters were delivered as late as after 43 days. Moreover, between the 78^th^ and 87^th^ day, the population declined to just a single adult male and two females (one additional pair was allowed to breed in the exit box, see above). After that, the population rapidly increased, and after 200 days, its size exceeded those of the other three runs (Fig. 1). In all experiments, the growth was interrupted by a few conspicuous drops in population size. These declines were caused mainly by the deaths of adult yet non-reproducing mice (Fig. S2).** Table S2 shows details on the total numbers of individuals, numbers of those that reached adulthood, and those participating in reproduction, as well as the number of delivered offspring and the number of litters). Table S3 provides information about the number of modules in four experimental populations, the lifespan of these modules, their size, and level of support for their demic nature.

**Table S2.** Total counts of individuals in four experimental populations. The first number refers to the first 210 days of the experiment (the length of the shortest, *domesticus* 2013, run), the number in brackets refers to the whole experiment.

|  | Total | Adults | Mothers | Fathers | Offspring | Litters | Mature |
| --- | --- | --- | --- | --- | --- | --- | --- |
| *domesticus* 2013 | 111 (111) | 68 (68) | 7 (7) | 4 (4) | 89 (89) | 17 (17) | 19 (19) |
| *domesticus* 2014 | 128 (186) | 42 (98) | 13 (19) | 9 (15) | 128 (192) | 27 (48) | 23 (35) |
| *musculus* 2013 | 120 (143) | 49 (100) | 11 (11) | 5 (5) | 91 (101) | 21 (25) | 23 (23) |
| *musculus* 2014 | 141 (268) | 79 (101) | 12 (22) | 13 (22) | 128 (229) | 27 (51) | 37 (49) |


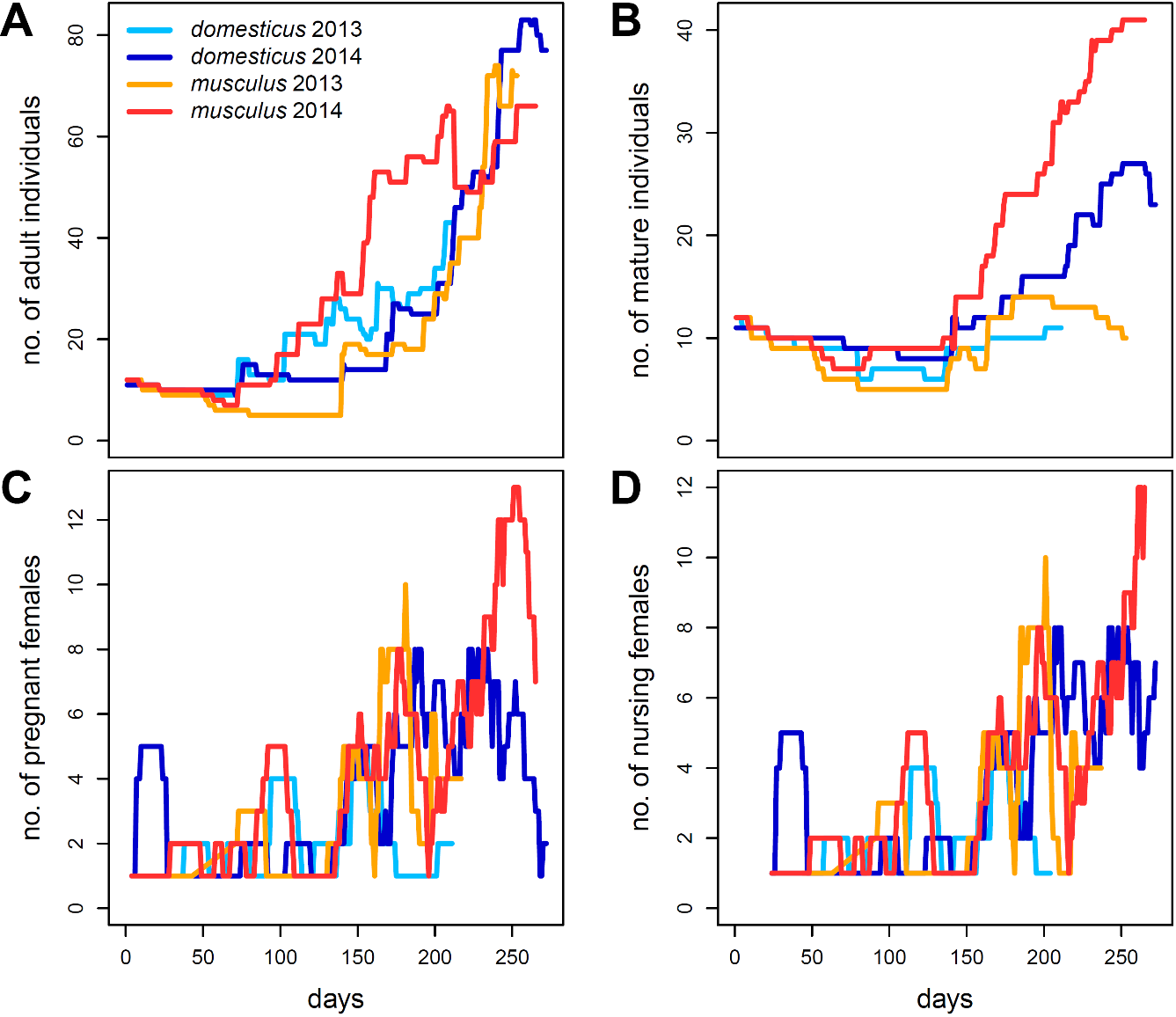


**Figure S2.** Changes in numbers of adult individuals (A), reproducing individuals (B), pregnant females (C), and nursing females (D). Individuals were considered to be adults since 50 days of age, whereas as reproducing, we considered mice within 20 days before the birth of their first offspring (with parentage proven by molecular analysis). As pregnant, we considered females within 20 days before parturition, and females within 20 days after delivery were taken as nursing.

**Table S3.** The number, lifespan, size, and support for the demic nature of modules in four experimental populations. The lifespan of modules is in days; in brackets, there are the first and last days of its existence (counted since the beginning of the experiment). The module size is the total number of individuals ever involved; in brackets, the total number of time layers in which the module existed, weighted by its layer-specific sizes.

|  | *domesticus* 2013 | | | *domesticus* 2014 | | | *musculus* 2013 | | | *musculus* 2014 | | |
| --- | --- | --- | --- | --- | --- | --- | --- | --- | --- | --- | --- | --- |
|  | Lifespan | Size | Deme | Lifespan | Size | Deme | Lifespan | Size | Deme | Lifespan | Size | Deme |
| 1 | 168 (1-168) | 7 (202) | 0.92 | 222 (1-222) | 19 (594) | 8.32 | 153 (1-153) | 2 (45) | 1.00 | 263 (1-263) | 99 (3223) | 33.04 |
| 2 | 210 (1-210) | 17 (542) | 3.91 | 122 (1-122) | 4 (144) | 3.45 | 70 (1-70) | 4 (69) | 0.00 | 263 (1-263) | 18 (792) | 3.61 |
| 3 | 210 (1-210) | 30 (1266) | 4.26 | 30 (53-82) | 3 (29) | 0.00 | 250 (1-250) | 120 (3040) | 23.00 | 158 (106-263) | 33 (804) | 3.44 |
| 4 | 21 (1-21) | 4 (22) | 0.00 | 164 (107-270) | 51 (1526) | 11.32 | 8 (1-8) | 2 (6) | 0.00 | 18 (141-158) | 1 (10) | 0.00 |
| 5 | 121 (90-210) | 22 (554) | 3.11 | 63 (107-169) | 6 (68) | 0.00 | 5 (1-5) | 4 (6) | 0.00 |  |  |  |
| 6 |  |  |  | 126 (145-270) | 51 (941) | 3.09 |  |  |  |  |  |  |
| 7 |  |  |  | 69 (178-246) | 3 (6) | 0.00 |  |  |  |  |  |  |
| 8 |  |  |  | 4 (182-185) | 3 (5) | 0.00 |  |  |  |  |  |  |
| 9 |  |  |  | 1 (199-199) | 3 (3) | 0.00 |  |  |  |  |  |  |
| 10 |  |  |  | 1 (239-239) | 2 (2) | 0.00 |  |  |  |  |  |  |
| 11 |  |  |  | 10 (259-268) | 6 (7) | 0.00 |  |  |  |  |  |  |

**Figure S3.** Bootstrap optimisation of the Infomap relax rate parameter. Violin plots show distributions of modularity values across bootstrap replicates as evaluated with particular relax rates (shown on the abscissa). The dots indicate modularity evaluated from the observed data.

**Figure S4.** Spatial separation quantified as a weighted mean of box usage exclusivities, calculated from all nest boxes that were in use in the particular time layer (which usually means all six boxes). The exclusivity of a box in a given time layer was defined as the maximum value of its possession by any deme.


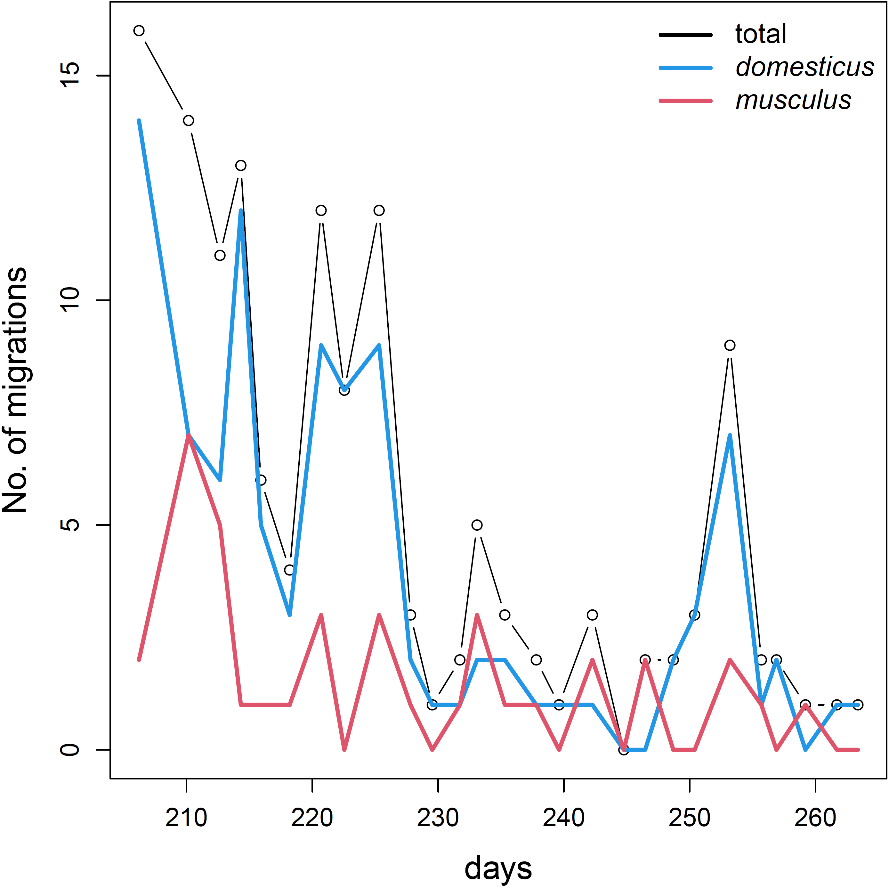


**Figure S5.** The number of migrations between enclosures after their connection in 2014 runs. We omitted five individuals accounting for nearly half of migrations in this figure. Of the total 266 migration events, 154 were due to just five males; the remaining 112 migrations were performed by 9 females/19 males of *domesticus* and 11 females/10 males of *musculus*.


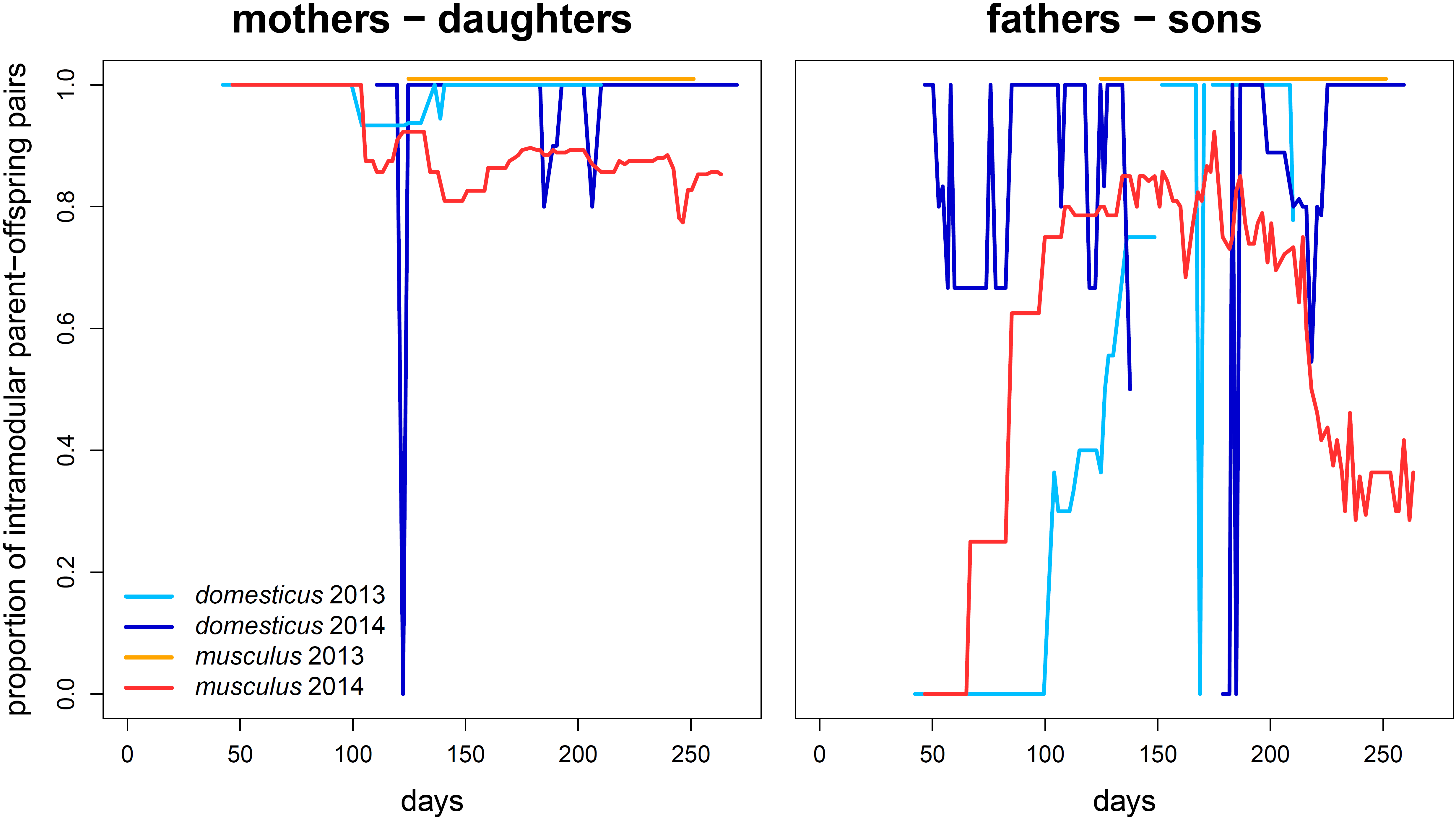


**Figure S6.** The persistence of family bonds within modules shown as changes in the proportion of mother-daughter and father-son pairs found within modules through time.

### Reference

1. Vyskočilová M, Trachtulec Z, Forejt J, Piálek J. 2005 Does geography matter in hybrid sterility in house mice?. *Biol. J. Linn. Soc*. **84**, 663–674. (doi:10.1111/j.1095-8312.2005.00463.x)
